# Associations between epilepsy-related polygenic risk and brain morphology in childhood

**DOI:** 10.1093/brain/awaf259

**Published:** 2025-08-14

**Authors:** Alexander Ngo, Lang Liu, Sara Larivière, Valeria Kebets, Serena Fett, Clara F Weber, Jessica Royer, Eric Yu, Raúl Rodríguez-Cruces, Zhiqiang Zhang, Leon Qi Rong Ooi, B T Thomas Yeo, Birgit Frauscher, Casey Paquola, Maria Eugenia Caligiuri, Antonio Gambardella, Luis Concha, Simon S Keller, Fernando Cendes, Clarissa L Yasuda, Leonardo Bonilha, Ezequiel Gleichgerrcht, Niels K N Focke, Raviteja Kotikalapudi, Terence J O’Brien, Benjamin Sinclair, Lucy Vivash, Patricia M Desmond, Elaine Lui, Anna Elisabetta Vaudano, Stefano Meletti, Reetta Kälviäinen, Hamid Soltanian-Zadeh, Gavin P Winston, Vijay K Tiwari, Barbara A K Kreilkamp, Matteo Lenge, Renzo Guerrini, Khalid Hamandi, Theodor Rüber, Tobias Bauer, Orrin Devinsky, Pasquale Striano, Erik Kaestner, Sean N Hatton, Lorenzo Caciagli, Matthias Kirschner, John S Duncan, Paul M Thompson, ENIGMA Consortium Epilepsy Working Group, Eugenio Abela, Julie Absil, Saud Alhusaini, Sarah J A Carr, Gianpiero L Cavalleri, Esmaeil Davoodi-Bojd, Norman Delanty, Chantal Depondt, Colin P Doherty, Martin Domin, Sonya Foley, Aoife Griffin, Graeme D Jackson, Magdalena Kowalczyk, Angelo Labate, Soenke Langner, Mario Mascalchi, Pascal Martin, Mark P Richardson, Christian Rummel, Mira Semmelroch, Mariasavina Severino, Aditi Singh, Rhys H Thomas, Manuela Tondelli, Domenico Tortora, Felix von Podewills, Sjoerd B Vos, Christopher D Whelan, Roland Wiest, Junsong Zhang, Carrie R McDonald, Sanjay M Sisodiya, Neda Bernasconi, Andrea Bernasconi, Ziv Gan-Or, Boris C Bernhardt

**Affiliations:** Montreal Neurological Institute and Hospital, McGill University, Montreal, QC, Canada H3A 2B4; Department of Neurology and Neurosurgery, McGill University, Montreal, QC, Canada H3A 1A1; Montreal Neurological Institute and Hospital, McGill University, Montreal, QC, Canada H3A 2B4; Department of Human Genetics, McGill University, Montreal, QC, Canada H3A 1Y2; Department of Medical Imaging and Radiation Sciences, Centre de Recherche du CHUS, Université de Sherbrooke, Sherbrooke, QC, Canada J1H 5N4; Montreal Neurological Institute and Hospital, McGill University, Montreal, QC, Canada H3A 2B4; Department of Neurology and Neurosurgery, McGill University, Montreal, QC, Canada H3A 1A1; Montreal Neurological Institute and Hospital, McGill University, Montreal, QC, Canada H3A 2B4; Department of Neurology and Neurosurgery, McGill University, Montreal, QC, Canada H3A 1A1; Montreal Neurological Institute and Hospital, McGill University, Montreal, QC, Canada H3A 2B4; Department of Psychiatry and Psychotherapy, University of Lübeck, Lübeck 23538, Germany; Centre of Brain, Behavior and Metabolism, University of Lübeck, Lübeck 23562, Germany; Montreal Neurological Institute and Hospital, McGill University, Montreal, QC, Canada H3A 2B4; Department of Neurology and Neurosurgery, McGill University, Montreal, QC, Canada H3A 1A1; Montreal Neurological Institute and Hospital, McGill University, Montreal, QC, Canada H3A 2B4; Department of Human Genetics, McGill University, Montreal, QC, Canada H3A 1Y2; Montreal Neurological Institute and Hospital, McGill University, Montreal, QC, Canada H3A 2B4; Department of Neurology and Neurosurgery, McGill University, Montreal, QC, Canada H3A 1A1; Department of Medical Imaging, Nanjing University School of Medicine, Nanjing 211166, China; Centre for Sleep and Cognition, National University of Singapore, Singapore 117549 Singapore; Centre for Translational Magnetic Resonance, National University of Singapore, Singapore 117549, Singapore; Department of Electrical and Computer Engineering, National University of Singapore, Singapore 117583, Singapore; Centre for Sleep and Cognition, National University of Singapore, Singapore 117549 Singapore; Centre for Translational Magnetic Resonance, National University of Singapore, Singapore 117549, Singapore; Department of Electrical and Computer Engineering, National University of Singapore, Singapore 117583, Singapore; Department of Neurology, Duke University, Durham, NC 27710, USA; Department of Biomedical Engineering, Duke University, Durham, NC 27708, USA; Institute of Neuroscience and Medicine (INM-7), Forschungszentrum Jülich, Jülich 52428, Germany; Neuroscience Research Center, University Magna Græcia, Catanzaro 88100, Italy; Institute of Neurology, University Magna Græcia, Catanzaro 88100, Italy; Institute of Neurobiology, Universidad Nacional Autónoma de México, Querétaro 76230, México; Institute of Systems, Molecular and Integrative Biology, University of Liverpool, Liverpool L69 7BE, UK; Walton Centre NHS Foundation Trust, Liverpool L9 7LJ, UK; Department of Neurology, University of Campinas–UNICAMP, Campinas, São Paulo 13083887, Brazil; Department of Neurology, University of Campinas–UNICAMP, Campinas, São Paulo 13083887, Brazil; Department of Neurology, University of South Carolina School of Medicine, Columbia, SC 29202, USA; Department of Neurology, Medical University of South Carolina, Charleston, SC 29425, USA; Department of Neurology, University of Medicine Göttingen, Göttingen 37075, Germany; Department of Neurology, University of Medicine Göttingen, Göttingen 37075, Germany; Department of Neuroscience, Central Clinical School, Alfred Hospital, Monash University, Melbourne, Melbourne, VIC 3004, Australia; Departments of Medicine and Radiology, The Royal Melbourne Hospital, University of Melbourne, Parkville, VIC 3050, Australia; Department of Neuroscience, Central Clinical School, Alfred Hospital, Monash University, Melbourne, Melbourne, VIC 3004, Australia; Departments of Medicine and Radiology, The Royal Melbourne Hospital, University of Melbourne, Parkville, VIC 3050, Australia; Department of Neuroscience, Central Clinical School, Alfred Hospital, Monash University, Melbourne, Melbourne, VIC 3004, Australia; Departments of Medicine and Radiology, The Royal Melbourne Hospital, University of Melbourne, Parkville, VIC 3050, Australia; Departments of Medicine and Radiology, The Royal Melbourne Hospital, University of Melbourne, Parkville, VIC 3050, Australia; Departments of Medicine and Radiology, The Royal Melbourne Hospital, University of Melbourne, Parkville, VIC 3050, Australia; Neurophysiology Unit and Epilepsy Centre, Azienda Ospedaliera-Universitaria, Modena 41126, Italy; Department of Biomedical, Metabolic and Neural Science, University of Modena and Reggio Emilia, Modena 41125, Italy; Neurophysiology Unit and Epilepsy Centre, Azienda Ospedaliera-Universitaria, Modena 41126, Italy; Department of Biomedical, Metabolic and Neural Science, University of Modena and Reggio Emilia, Modena 41125, Italy; Epilepsy Center, Neuro Center, Kuopio University Hospital, Member of the European Reference Network for Rare and Complex Epilepsies EpiCARE, Kuopio 70210, Finland; Faculty of Health Sciences, School of Medicine, Institute of Clinical Medicine, University of Eastern Finland, Kuopio 70210, Finland; Control and Intelligent Processing Center of Excellence (CIPCE), School of Electrical and Computer Engineering, University of Tehran, Tehran 1439957131, Iran; Departments of Research Administration and Radiology, Henry Ford Health System, Detroit 48202, USA; Division of Neurology, Department of Medicine, Queen’s University, Kingston, ON, Canada K7L 2V7; Department of Epilepsy, UCL Queen Square Institute of Neurology, London WC1N 3BG, UK; Chalfont Centre for Epilepsy, Epilepsy Society, Bucks SL9 0RJ, UK; Institute for Molecular Medicine, University of Southern Denmark, Odense s5230, Denmark; Department of Neurology, University of Medicine Göttingen, Göttingen 37075, Germany; Neuroscience and Human Genetics Department, Meyer Children’s Hospital IRCCS, Florence 50139, Italy; Neuroscience and Human Genetics Department, Meyer Children’s Hospital IRCCS, Florence 50139, Italy; Department of Neuroscience, Psychology, Drug Research and Child Health, University of Florence, Florence 50121, Italy; The Wales Epilepsy Unit, Department of Neurology, University Hospital of Whales, Cardiff CF14 4XW, UK; Cardiff University Brain Research Imaging Centre (CUBRIC), College of Biomedical Sciences, Cardiff University, Cardiff CF24 4HQ, UK; Department of Epileptology, University Hospital Bonn, Bonn 53127, Germany; Department of Neuroradiology, University Hospital Bonn, Bonn 53127, Germany; German Center for Neurodegenerative Diseases (DZNE), Bonn 53127, Germany; Center for Medical Data Usability and Translation, University of Bonn, Bonn 53113, Germany; Department of Epileptology, University Hospital Bonn, Bonn 53127, Germany; Department of Neuroradiology, University Hospital Bonn, Bonn 53127, Germany; German Center for Neurodegenerative Diseases (DZNE), Bonn 53127, Germany; Department of Neurology, NYU Grossman School of Medicine, New York, NY 10017, USA; Department of Neurosciences, Rehabilitation, Ophthalmology, Genetics, Maternal and Child Health, University of Genova, Genova 16147, Italy; IRCCS Instituto Giannina Gaslini, full member of ERN EpiCare, Genova 16147, Italy; Department of Radiation Medicine and Applied Sciences, University of California San Diego, La Jolla, CA 92093, USA; Department of Neurosciences, Center for Multimodal Imaging and Genetics, University of California San Diego, La Jolla, CA 92093, USA; Division of Neurology, Department of Medicine, Queen’s University, Kingston, ON, Canada K7L 2V7; Department of Neurology, Inselspital, Sleep-Wake-Epilepsy-Center, Bern University Hospital, University of Bern, Bern 3010, Switzerland; Department of Psychiatry, Psychotherapy and Psychosomatics, Psychiatric Hospital University of Zurich, Zurich 8008, Switzerland; Division of Adult Psychiatry, Department of Psychiatry, Geneva University Hospitals, Geneva 1205, Switzerland; Division of Neurology, Department of Medicine, Queen’s University, Kingston, ON, Canada K7L 2V7; Department of Epilepsy, UCL Queen Square Institute of Neurology, London WC1N 3BG, UK; Imaging Genetics Center, Mark & Mary Stevens Institute for Neuroimaging and Informatics, Keck School of Medicine, University of Southern California, Los Angeles, CA 90033, USA; Department of Radiation Medicine and Applied Sciences, University of California San Diego, La Jolla, CA 92093, USA; Department of Psychiatry, Center for Multimodal Imaging and Genetics, University of California San Diego, La Jolla, CA 92093, USA; Division of Neurology, Department of Medicine, Queen’s University, Kingston, ON, Canada K7L 2V7; Department of Epilepsy, UCL Queen Square Institute of Neurology, London WC1N 3BG, UK; Montreal Neurological Institute and Hospital, McGill University, Montreal, QC, Canada H3A 2B4; Department of Neurology and Neurosurgery, McGill University, Montreal, QC, Canada H3A 1A1; Montreal Neurological Institute and Hospital, McGill University, Montreal, QC, Canada H3A 2B4; Department of Neurology and Neurosurgery, McGill University, Montreal, QC, Canada H3A 1A1; Montreal Neurological Institute and Hospital, McGill University, Montreal, QC, Canada H3A 2B4; Department of Neurology and Neurosurgery, McGill University, Montreal, QC, Canada H3A 1A1; Department of Human Genetics, McGill University, Montreal, QC, Canada H3A 1Y2; Montreal Neurological Institute and Hospital, McGill University, Montreal, QC, Canada H3A 2B4; Department of Neurology and Neurosurgery, McGill University, Montreal, QC, Canada H3A 1A1

**Keywords:** imaging-genetics, temporal lobe epilepsy, brain structure, genetic risk, childhood

## Abstract

Extensive neuroimaging research in temporal lobe epilepsy with hippocampal sclerosis (TLE-HS) has identified brain atrophy as a disease phenotype. While it is also related to a complex genetic architecture, the transition from genetic risk factors to brain vulnerabilities remains unclear. Using a population-based approach, we examined the associations between epilepsy-related polygenic risk for HS (PRS-HS) and brain structure in healthy developing children, assessed their relation to brain network architecture, and evaluated its correspondence with case-control findings in TLE-HS diagnosed patients relative to healthy individuals.

We used genome-wide genotyping and structural T1-weighted MRI of 3826 neurotypical children from the Adolescent Brain Cognitive Development (ABCD) study. Surface-based linear models related PRS-HS to cortical thickness measures, and subsequently contextualized findings with structural and functional network architecture based on epicentre mapping approaches. Imaging-genetic associations were then correlated to atrophy and disease epicentres in 785 patients with TLE-HS relative to 1512 healthy controls aggregated across multiple sites.

Higher PRS-HS was associated with decreases in cortical thickness across temporo-parietal as well as fronto-central regions of neurotypical children. These imaging-genetic effects were anchored to the connectivity profiles of distinct functional and structural epicentres. Compared with disease-related alterations from a separate epilepsy cohort, regional and network correlates of PRS-HS strongly mirrored cortical atrophy and disease epicentres observed in patients with TLE-HS and were highly replicable across different studies. Findings were consistent when using statistical models controlling for spatial autocorrelations and robust to variations in analytic methods.

Capitalizing on recent imaging-genetic initiatives, our study provides novel insights into the genetic underpinnings of structural alterations in TLE-HS, revealing common morphological and network pathways between genetic vulnerability and disease mechanisms. These signatures offer a foundation for early risk stratification and personalized interventions targeting genetic profiles in epilepsy.

## Teaser

Temporal lobe epilepsy has a complex genetic basis, but how genetic vulnerability leads to brain changes is unclear. Ngo *et al*. show that higher risk for TLE in healthy children is linked to brain thinning in key regions that mirror patterns in patients. These findings help connect genetics to brain changes and may aid early risk detection.

## Introduction

Epilepsy is characterized by an enduring predisposition to recurrent spontaneous seizures and affects over 50 million people worldwide.^1^ One of the most common forms of epilepsy is temporal lobe epilepsy (TLE), a focal epilepsy associated pathologically with hippocampal sclerosis (HS) and pharmacoresistance. Cumulative evidence has underscored the complexity of TLE-HS, revealing contributions from genetic and acquired factors in epileptogenesis. With seizure onsets typically in childhood and adolescence,^2^ developmental transitions spanning youth represent a key window for epilepsy risk. Adequately capturing the condition's effects on brain organization, particularly in development, may advance our understanding of brain mechanisms giving rise to seizures and may have important implications for disease monitoring and early diagnosis.

In addition to its typical association with mesiotemporal pathology, neuroimaging evidence in patients with TLE-HS has identified widespread structural alterations. MRI analysis of brain morphology has established robust structural compromise in the hippocampus, subcortical regions, as well as more widespread temporal and fronto-central cortical systems. These findings were initially shown in single-centre studies,^3-6^ and more recently confirmed in large-scale multisite consortia, notably ENIGMA-Epilepsy (Enhancing Neuro Imaging Genetics through Meta Analysis-Epilepsy Consortium).^7,8^ The latter initiative has mapped consistent patterns of multilobar atrophy in TLE-HS, and further contextualized findings with measures of brain network architecture, confirming temporo-limbic regions as epicentres of distributed structural pathology.^9^ Despite a likely influence of environmental factors and clinical events on brain structure in TLE,^10^ there has been growing evidence of important genetic influence,^11^ suggesting a possible mechanism affecting this classical disease phenotype.

Epilepsy has a complex genetic architecture, with many contributory genetic factors.^12-16^ Variants underlying many different monogenic forms of epilepsy are rare, yet of large effect that can confer high risk or be causally responsible for the disease.^17,18^ Despite the clinical implications of these variants, common epilepsy syndromes, particularly TLE-HS, rarely carry such variants and presumably have a complex, multigenic inheritance.^19^ Causation may therefore be attributable to the synergy of multiple genetic variants interacting with each other, together with acquired environmental factors. Recent genome-wide association studies (GWAS) have identified common risk alleles.^13-16^ These individual genetic risk variants are usually of small effect and cannot quantify risk or inform prognosis and treatment.^20^ However, genome-wide profiling using polygenic risk scores (PRS) may provide a window into the genetic liability of the disease. By estimating the combined effect of individual single nucleotide polymorphisms (SNPs), it can collectively capture the variance explained by these common alleles and provide an individualized measure of genetic risk.^21-23^ While previous studies have revealed enriched genetic vulnerability for epilepsy in patients,^24-26^ the consequences of epilepsy susceptibility on disease phenotypes, such as brain morphology, have not been systematically charted. Investigating this micro-to-macroscale mechanism may provide insight into the translation of genetic vulnerability to disease aetiology or consequences.

**Figure 1:**
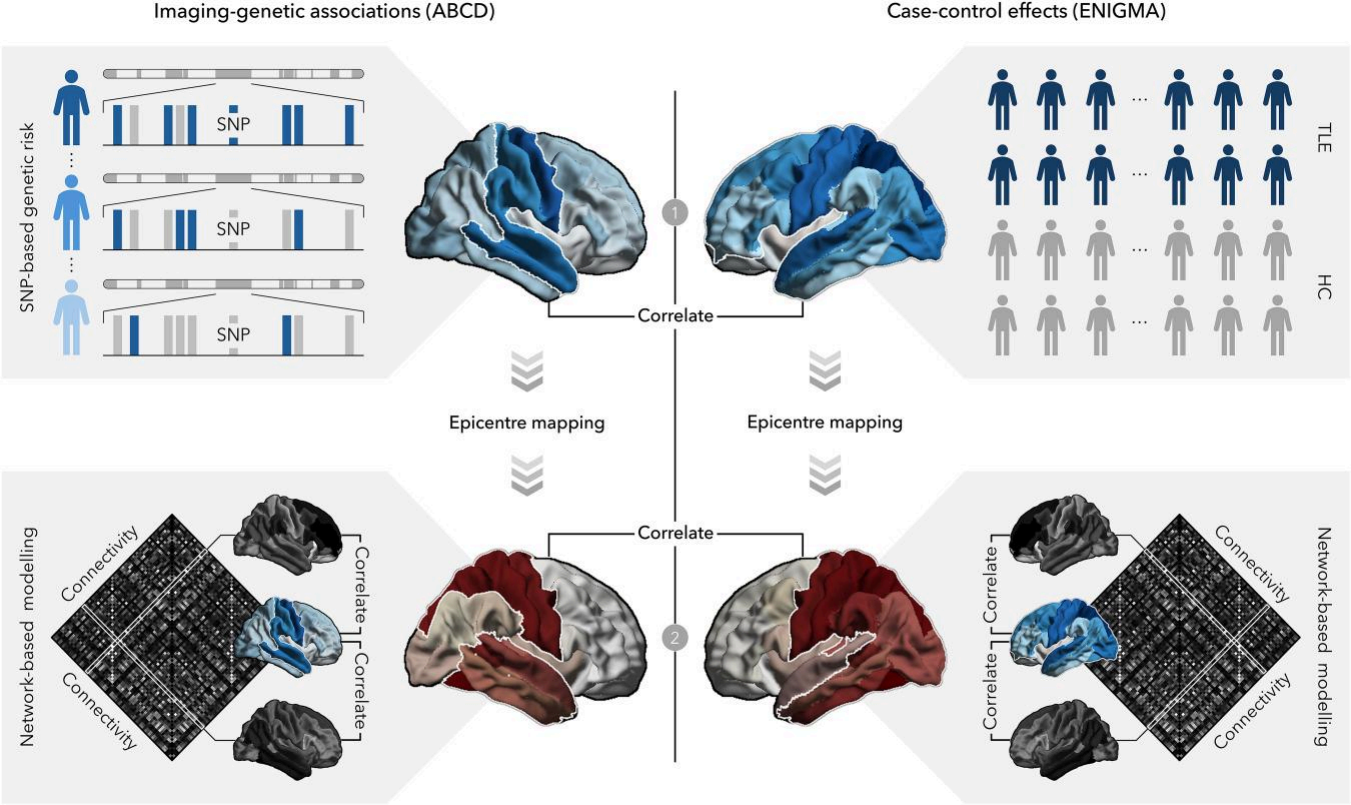
**Overview of the study design**. Regional and epicentre profiles of imaging-genetic associations (*left*) are correlated to disease effects observed in case-control studies *(right*). HC = healthy control; SNP = single nucleotide polymorphism; TLE-HS = temporal lobe epilepsy with hippocampal sclerosis.

In this study, we aimed to uncover the cumulative effects of epilepsy-related genetic risk variants on structural brain organization during development. We analysed structural MRI and genotyping data in a large population-based cohort of neurotypical children from the Adolescent Brain Cognitive Development study (ABCD).^27^ To investigate associations between genetic risk factors for epilepsy-related HS and brain-wide morphology, we generated PRS-related models of cortical thickness and subcortical volume. Network contextualization further identified connectome epicentres of PRS-HS effects—network pathways that may govern the genetically affected morphological patterning. To pinpoint common processes between genetic risk and disease pathologies, we employed spatial correlations with autocorrelation preserving null models and related structural effects of PRS-HS to disease-related atrophy and epicentres derived from large multi-site MRI-based datasets of patients and controls (Fig. 1).^28-30^

## Materials and methods

### Participants

#### ABCD study

The present study used the demographic, genetic and neuroimaging data of 3826 unrelated neurotypical children [mean ± standard deviation (SD), age = 10.0 ± 0.6 years; 2052 males] from the multisite ABCD 2.0.1 release^31^ and selected based on the availability of high-quality T1-weighted MRI and genome-wide genotyping data, as well as European genetic ancestry (described in the subsequent sections). Briefly, participants were recruited based on probability sampling of schools near the study sites. Parents or guardians provided written consent, while the child provided written assent. All aspects of the ABCD study were approved by the Institutional Review Board at the University of California, San Diego, USA. Overall, the large size of this cohort allows for unprecedented exploration of genetic risk for TLE-HS and its potential effects on brain organization in an *a priori* neurotypical child population.

#### Human Connectome Project

We also selected 50 unrelated healthy adults from the Human Connectome Project (HCP) dataset (imaging acquisition and processing are described in the Supplementary material).^32^ Such initiatives provide normative structural and functional connectivity information to employ network epicentre mapping of PRS-HS.

#### ENIGMA-Epilepsy Consortium

Imaging-genetic associations from neurotypical children were compared to MRI-based disease effects observed between 732 patients with TLE and radiological evidence of HS (mean ± SD, age = 38.6 ± 10.6 years; 329 males; 391 left-sided focus) and 1418 (mean ± SD, age = 33.8 ± 10.5 years; 643 males) healthy controls (HC). Details of case-control cohorts are described in the Supplementary material and elsewhere.^28^

#### Independent temporal lobe epilepsy with hippocampal sclerosis case-control datasets

To assess the replication of the aforementioned analysis, imaging-genetic associations were also compared to structural alterations observed between 53 individuals with pharmaco-resistant TLE-HS and 93 age- (*t* = 1.51, *P* = 0.13) and sex-matched (*χ*^2^ = 0.13, *P* = 0.72) HCs. Case-control participants were selected from (i) Montreal Neurological Institute and Hospital (MICs; *n*_TLE-HS/HC_ = 23/36)^29^; and (ii) Jinling Hospital (NKG; *n*_TLE-HS/HC_ = 37/57).^30^ Sociodemographic, clinical and imaging details of the two sites are in the Supplementary material.

### Genomic data acquisition and processing of ABCD study data

#### Single nucleotide polymorphism genotyping

A total of 550 000 SNPs were genotyped from saliva samples using the Affymetrix Axiom Smokescreen Array platform.^33,34^ The data were prepared for imputation using ‘imputePrepSanger’ pipeline (https://hub.docker.com/r/eauforest/imputeprepsanger/), implemented on CBRAIN^35^ and the Human660W-Quad_v1_A-b37-strand chip as reference.

#### Genotyping quality control and imputation

Genotyping was quality controlled using PLINK 1.9.^36^ Steps included: (i) assessment of heterozygosity using the PLINK–indep-pairwise command with parameters set to 200, 50 and 0.15; (ii) removal of samples whose heterozygosity *F* coefficient was greater than 3 SD units from the mean; (iii) removal of samples and SNPs with low call rate at 0.01 and all SNPs with minor allele frequency (MAF) < 0.01; (iv) removal of individuals with mismatched sex and gender; (v) exclusion of non-European individuals by principal component analysis (PCA) with Hapmap; (vi) removal of samples with a first- or second-degree relative in the cohort (π > 0.125); (vii) application of a haplotype-based test for non-random missing genotype data to remove SNPs at *P* < 1× 10^–4^ where they had non-random associations between unobserved genotypes and missingness; and (viii) application of a test for Hardy-Weinberg equilibrium (HWE) and removal of SNPs significant at *P* < 1 × 10^−6^. Imputation was performed using the Michigan Imputation Service with the Haplotype Reference Consortium (HRC) r1.1 2016 (hg19) as a reference panel.^37^

#### Deriving polygenic risk scores

Individualized PRS were computed using the summary statistics from an epilepsy genome-wide association study for focal epilepsy with documented HS.^15^ While this may not necessarily equate to TLE-HS, we used this classification as a close proxy given the high prevalence and relative specificity of HS in TLE. SNPs with an INFO < 0.8 and an MAF < 0.01 were excluded, and duplicate SNPs were removed. PRSice-2 was used to calculate genetic risk scores.^38^ Given that an optimal probability threshold (*P*_SNP_) related to HS was not previously reported, we used multiple *P*_SNP_ that significantly predicted focal epilepsy: 0.001, 0.05, 0.1, 0.2, 0.3, 0.4, 0.5.^26^ All main analyses used PRS constructed at *P*_SNP_ < 0.1, with consistency of findings evaluated across remaining thresholds.

### Imaging acquisition and processing of ABCD study cohort

#### Acquisition

All participants underwent 3T MRI scanning with prospective motion correction to reduce head motion and distortions, including a 3D T1-weighted anatomical scan based on a magnetization-prepared rapid acquisition gradient echo sequence.^31^

#### Processing

T1-weighted data were processed using FreeSurfer (version 5.3.0) to generate cortical surface and subcortical segmentations.^39,40^ Based on the Desikan-Killiany anatomical atlas,^41^ subject-specific maps of cortical thickness were sampled across 68 grey matter brain regions, and volume measures were obtained from 12 subcortical grey matter regions (bilateral amygdala, caudate, nucleus accumbens, pallidum, putamen and thalamus) and bilateral hippocampi.

#### Multisite data harmonization

Morphological data were harmonized across sites using ComBat (https://github.com/Jfortin1/ComBatHarmonization), a post-acquisition statistical batch normalization of between-site effects, while preserving age, sex and genetic risk.^42^

### Statistical analyses

#### Structural correlates of polygenic risk for hippocampal sclerosis

We implemented surface-based linear models in BrainStat (version 0.4.2; https://brainstat.readthedocs.io/)^43^ with age, sex, and the first 10 genetic principal components as covariates, similar to previous imaging-genetics studies.^44-46^ These related PRS-HS to cortical thickness and subcortical volume in neurotypical children from ABCD. Multiple comparisons were then corrected using the false discovery rate (FDR) procedure.^47^

To assess potential hemispheric asymmetry in the association between PRS-HS and cortical morphology, we computed interhemispheric asymmetric indices for thickness across homologous regions: AI=(left−right)/|(left+right)/2|, where AI is asymmetry index and left and right are the cortical thickness of left and right areas. Correlations between asymmetry and PRS-HS were assessed using similar linear models.

#### Network substrates of polygenic risk-related structural changes

We identified morphological polygenic risk epicentres by spatially correlating each brain region's healthy functional and structural connectivity profiles from the HCP dataset to the imaging-genetic map (i.e. the unthresholded *t*-statistic map from the above analysis). This approach was repeated systematically across all cortical and subcortical regions with non-parametric spin permutation null models to control for spatial autocorrelation (5000 repetitions),^48^ implemented in the ENIGMA toolbox (version 2.0.3; https://enigma-toolbox.readthedocs.io/).^49^ Higher spatial similarity between a given node's connectivity profile and whole-brain patterns of PRS-HS vulnerability supported that the node was an epicentre.

Dissociating the effects of network architecture from potential confounds introduced in normative connectomes, we also generated PRS-related epicentres using TLE-specific structural and functional connectomes (image processing and connectivity computations are described in the Supplementary material).

#### Relation to disease-specific atrophy and network epicentres

We identified the spatial overlap between imaging-genetic correlates from ABCD and epilepsy-related alterations. The latter were obtained previously published statistical case-control atrophy and epicentre maps for left and right TLE-HS from ENIGMA-Epilepsy.^8,28^ Spin permutation-based testing (5000 repetitions) assessed significant spatial associations between imaging-genetic and case-control effects at the regional and network levels.

We furthermore performed spatial correlations with case-control atrophy and epicentre maps for left and right TLE-HS from independent case-control datasets (MICs and NKG). Patient-specific morphology maps were *z*-scored relative to controls. We then used surface-based linear models with age, sex, and site as covariates to compare between groups. Subsequent epicentre analysis was performed on the TLE-HS atrophy profile. Spin permutation-based testing (5000 repetitions) evaluated significant spatial correlations between imaging-genetic and case-control effects.^48,49^

To evaluate the specificity of imaging-genetic effects to TLE-HS, we repeated the same analyses with idiopathic generalized epilepsy (IGE), another common epilepsy syndrome,^28^ and six psychiatric disorders [attention deficit hyperactivity disorder (ADHD), autism spectrum disorder (ASD), bipolar disorder (BD), major depressive disorder (MDD), obsessive-compulsive disorder (OCD) and schizophrenia (SCZ)], all acquired from the ENIGMA Consortium.^49,50^ Correlation coefficients were statistically compared to those observed in TLE-HS using Fisher z-transformation. Significance testing of these correlations and their differences was assessed using spin permutation tests with 5000 repetitions.^48,49^

#### Transcriptomic associations

To investigate the molecular pathways that may link cortical vulnerability to disease atrophy, regional imaging-genetic and case-control patterns were related with gene expression derived from the ENIGMA toolbox,^49^ which aggregates preprocessed post-mortem bulk microarray data from the Allen Human Brain Atlas.^51^ For each available gene (*n*_total_ = 12 668), we computed the spatial correlation between regional expression and imaging phenotype of interest (i.e. PRS-mediated thinning and left/right TLE-HS atrophy). Based on autocorrelation-preserving null models (*n* = 5000),^48,49^ we identified significantly correlated genes for both maps, and subsequently their intersection. A gene ontology enrichment analysis (https://www.webgestalt.org) was utilized to uncover biological processes enriched in the list of shared genes.^52^

#### Robustness analyses

To verify that results were not biased by choosing a particular threshold, we repeated the PRS analyses and associations with case-control atrophy across all predictive P_SNP_ thresholds (0.001, 0.05, 0.1, 0.2, 0.3, 0.4, 0.5).^26^ Specifically, PRS-HS was constructed at each threshold, and spatial correlations between all pairs of imaging-genetic brain maps were performed. Spin permutation-based testing (5000 repetitions) evaluated significant spatial correlations between imaging-genetic and case-control effects.^48,49^

## Results

### Structural correlates of polygenic risk for hippocampal sclerosis

**Figure 2:**
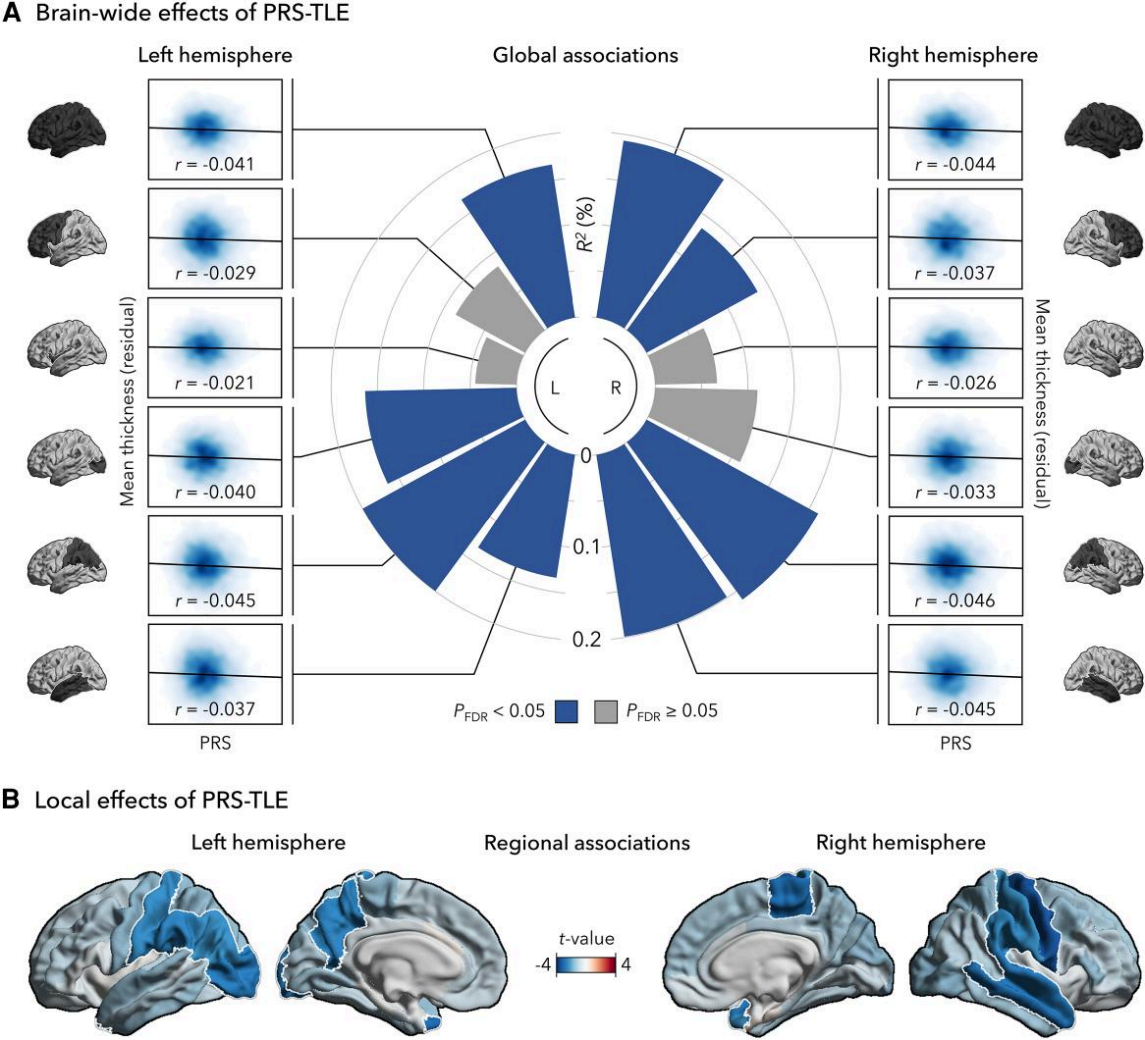
**PRS-HS associations with cortical thickness (ABCD)**. (**A**) Distribution of genetic risk effects on morphology across the different lobes (in order from top to bottom: all, frontal, limbic, occipital, parietal, temporal). (**B**) Regional imaging-genetic correlations between PRS-HS and thickness. Blue and red colours represent negative and positive correlations, respectively. White outline indicates *P*_FDR_ < 0.05. ABCD = Adolescent Brain Cognitive Development study; FDR = false discovery rate; L = left; PRS-HS = polygenic risk score for epilepsy-related hippocampal sclerosis; R = right.

We observed a significant and negative association between global cortical thickness and genetic vulnerability (left hemisphere: Pearson's correlation coefficient *r* = −0.041, *P*_FDR_ < 0.05; right hemisphere: *r* = −0.044, *P*_FDR_ < 0.05; Fig. 2A). Adopting a regional approach, these effects colocalized to bilateral temporal pole and postcentral gyrus, left precuneus, inferior parietal and lateral occipital regions as well as right superior and middle temporal, precentral and paracentral gyri (range *r* = −0.0501 – −0.0362, *P*_FDR_ < 0.05; Fig. 2B).

After correcting for multiple comparisons, no significant relationships between PRS-HS and subcortical and hippocampal volume (all *P*_FDR_ ≥ 0.05; Supplementary Fig. 1), as well as morphologically-related asymmetry were observed (all *P*_FDR_ ≥ 0.05; Supplementary Fig. 2).

### Network substrates of polygenic risk-related structural changes

**Figure 3:**
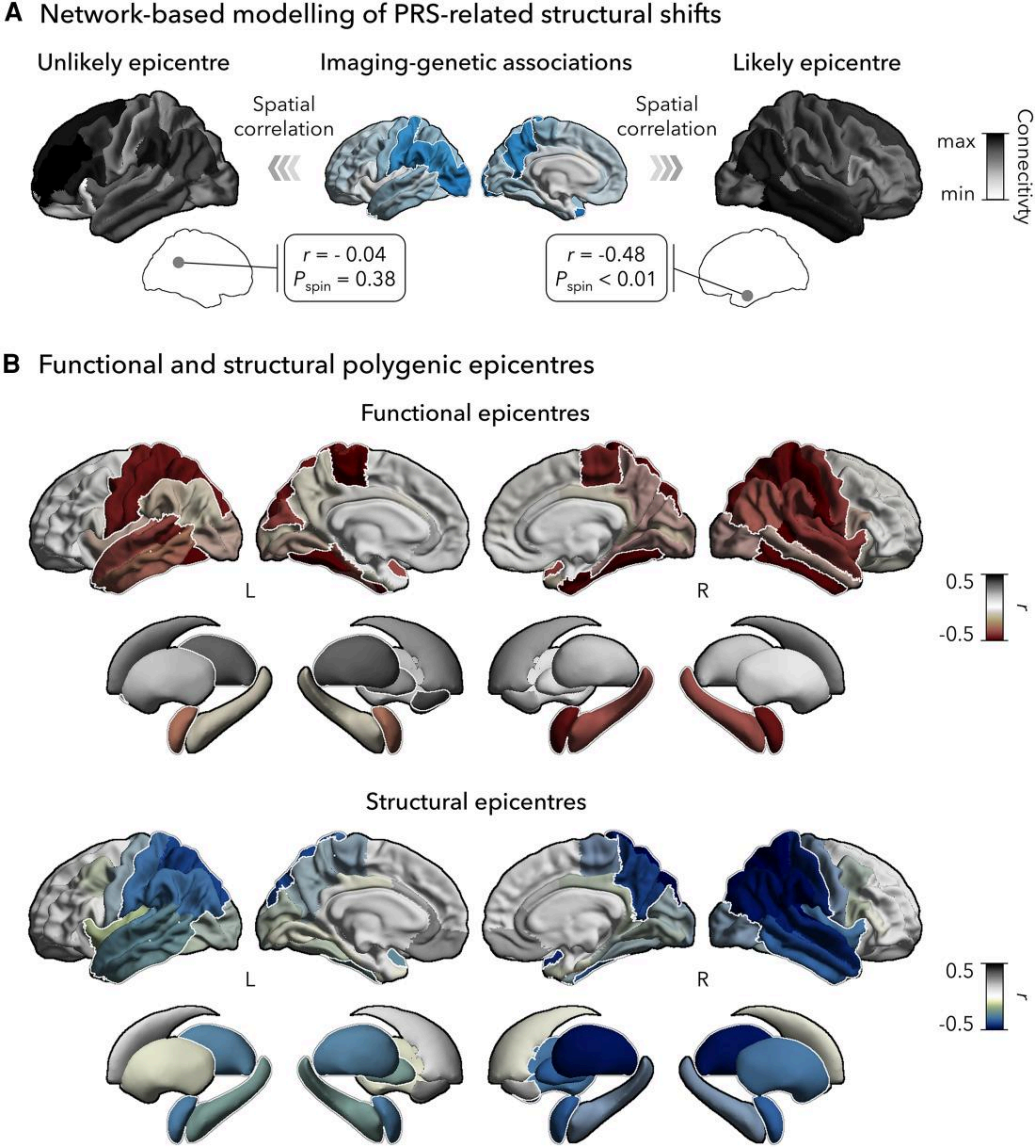
**Network epicentres of morphological changes associated with PRS-HS**. (**A**) Schematic representation of epicentre mapping approach using seed-based cortico- and subcortico-cortical connectivity. (**B**) Correlation coefficients indexing spatial similarity between imaging-genetic effects and seed-based functional (*top*) and structural (*bottom*) connections for every cortical and subcortical region. Red and blue colours represent negative associations, while grey depicts positive correlations. White outline indicates *P*_spin_ < 0.05. L = left; PRS-HS = polygenic risk score for epilepsy-related hippocampal sclerosis; R = right; spin = Spin permutation-based testing.

Given the large-scale effects of PRS-HS on cortical thickness, contextualizing imaging-genetic correlations with connectome architecture may provide insight into how localized genetic susceptibility propagates through distributed brain networks and predicts structural vulnerabilities. We systematically correlated imaging-genetic patterns (see Fig. 2) with the functional and structural connections of each cortical and subcortical region (Fig. 3A).^48^ This implicated bilateral temporal-limbic and parietal cortices, amygdalae, hippocampi, and thalami as the most significant functional and structural epicentres (all *P*_spin_ < 0.05; Fig. 3B).

Network profiles were also similar when using TLE-specific connectomes (functional: *r* = 0.86, *P*_spin_ < 0.001; structural: *r* = 0.98, *P*_spin_ < 0.001; Supplementary Fig. 3).

### Relation to epilepsy-specific atrophy and network epicentres

**Figure 4:**
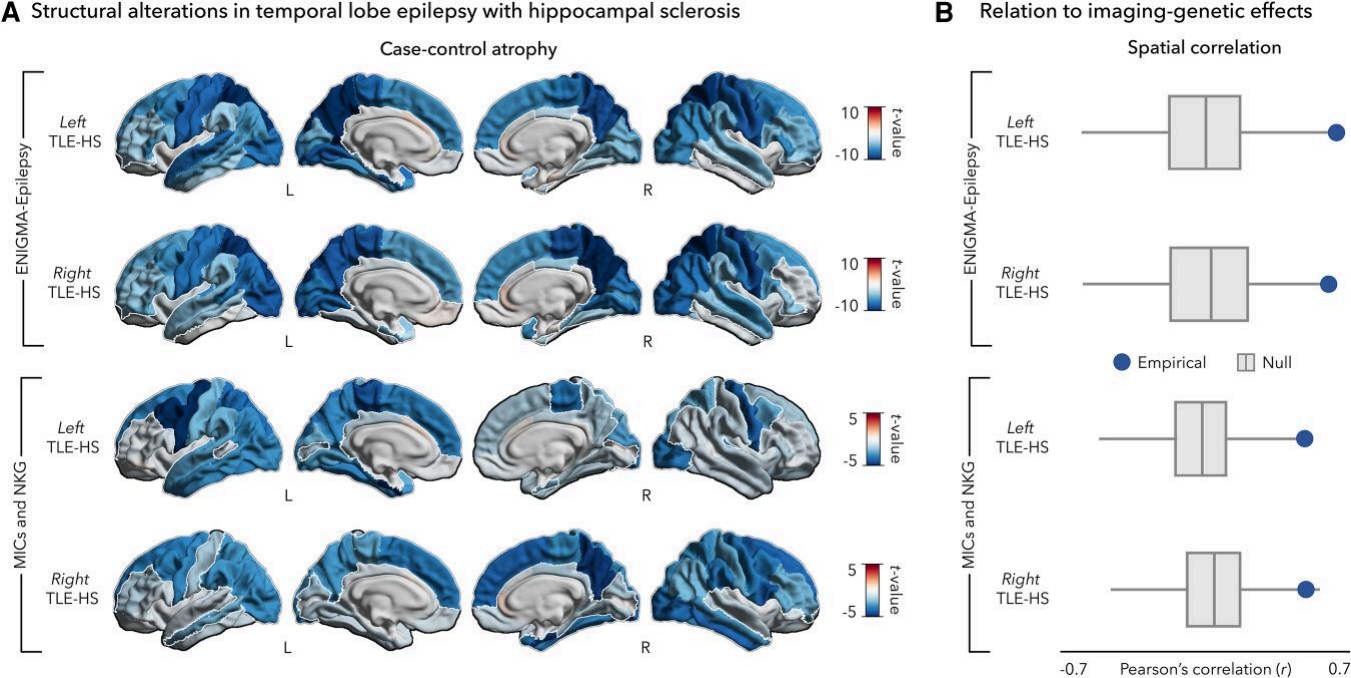
**Comparison between PRS-HS effects and epilepsy case-control atrophy**. (**A**) Case-control differences in left and right TLE-HS from ENIGMA-Epilepsy (*top*) and from MICs and NKG (*bottom*). Blue and red colours point to atrophy and hypertrophy in patients relative to healthy controls, respectively. Outline in white represents *P*_FDR_ < 0.05. L = left; R = right; TLE-HS = temporal lobe epilepsy with hippocampal sclerosis. (**B**) Spatial correlations between epilepsy-related atrophy (*top*: ENIGMA-Epilepsy; *bottom*: MICs and NKG) and imaging-genetic effect maps (ABCD) are compared against permutation-based null correlations. Points represent the empirical correlation (with significance defined as *P*_spin_ < 0.05). In the box plots, the ends of boxes represent the first (25%) and third (75%) quartiles, the centre line (median) represents the second quartile of the null distribution (*n* = 5000 permutations) and the whiskers represent the non-outlier end points of the distribution. ABCD = Adolescent Brain Cognitive Development study; ENIGMA-Epilepsy = Enhancing Neuro Imaging Genetics through Meta Analysis-Epilepsy Consortium; FDR = false discovery rate; MICs = Montreal Neurological Institute and Hospital; NKG = Jinling Hospital; spin = Spin permutation-based testing.

To link genetic vulnerability to disease alterations, we examined the spatial resemblance between imaging-genetic findings to atrophy patterns observed in individuals with TLE-HS. Assessing structural alterations in patients relative to controls (ENIGMA-Epilepsy), profound atrophy was observed, with the strongest effects in bilateral precuneus, precentral, paracentral and temporal cortices (*P*_FDR_ < 0.05; Fig. 4A). Correlating alteration maps with PRS effects (from ABCD, see Fig. 1) showed significant overlap with left (*r* = 0.63, *P*_spin_ = 0.001) and right TLE-HS (*r* = 0.59, *P*_spin_ = 0.0006; Fig. 4B).

**Figure 5:**
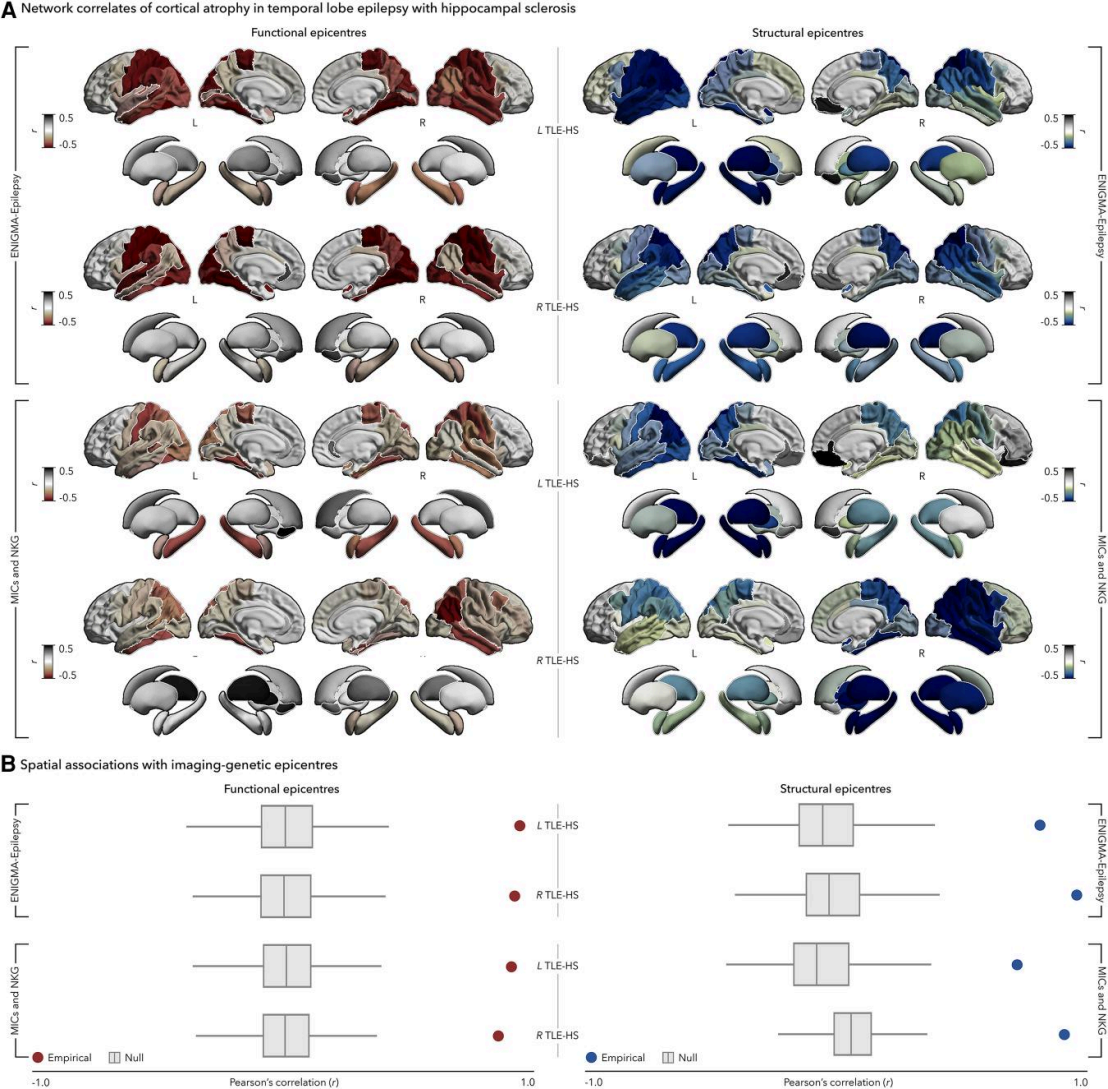
**Comparison between imaging-genetic and epilepsy-related disease epicentres.** (**A**) Functional and structural disease epicentres in left and right TLE-HS from ENIGMA-Epilepsy (*top*) and from MICs and NKG (*bottom*). Red and blue colours represent negative associations, while grey depicts positive correlations. Outline in white represents *P*_spin_ < 0.05. L = left; R = right; TLE-HS = temporal lobe epilepsy with hippocampal sclerosis. (**B**) Spatial correlations between epilepsy-related (*top*: ENIGMA-Epilepsy; *bottom*: MICs and NKG) and imaging-genetic epicentre maps (ABCD) are compared against permutation-based null correlations. Points represent the empirical correlation (with significance defined as *P*_spin_ < 0.05). In the box plots, the ends of boxes represent the first (25%) and third (75%) quartiles, the centre line (median) represents the second quartile of the null distribution (*n* = 5000 permutations) and the whiskers represent the non-outlier end points of the distribution. ABCD = Adolescent Brain Cognitive Development study; ENIGMA-Epilepsy = Enhancing Neuro Imaging Genetics through Meta Analysis-Epilepsy Consortium; MICs = Montreal Neurological Institute and Hospital; NKG = Jinling Hospital; spin = Spin permutation-based testing.

Network mapping of atrophy (ENIGMA-Epilepsy) revealed significant temporo-limbic and parieto-occipital epicentres in TLE-HS (*P*_FDR_ < 0.05; Fig. 5A). Similarly, imaging-genetic epicentres (from ABCD, see Fig. 2) were strongly correlated with disease epicentres in left TLE-HS (functional: *r* = 0.95, *P*_spin_ < 0.001; structural: *r* = 0.78, *P*_spin_ < 0.001) and right TLE-HS (functional: *r* = 0.93, *P*_spin_ < 0.001; structural: *r* = 0.94, *P*_spin_ < 0.001; Fig. 5B), suggesting potential pathway convergence between PRS-HS and TLE-HS effects.

These region- and network-level correlations were highly consistent when correlating PRS effects (from ABCD, see Figs 1 and 2) with separate, independent patient-control sites (MICs, NKG). Comparison between PRS effects (from ABCD, see Fig. 1) and disease-related atrophy (Fig. 4A) revealed moderate and highly significant positive correlations for left (*r* = 0.50, *P*_spin_ = 0.0002) and right TLE-HS (*r* = 0.41, *P*_spin_ = 0.009; Fig. 4B). Imaging-genetic epicentres (from ABCD, see Fig. 2) were also strongly similar with disease epicentres (Fig. 5A) in left (functional: *r* = 0.93, *P*_FDR_ < 0.001; structural: *r* = 0.77, *P*_FDR_ < 0.001) and right TLE-HS (functional: *r* = 0.89, *P*_FDR_ < 0.001; structural: *r* = 0.89, *P*_FDR_ < 0.001; Fig. 5B).

Cross-referencing our imaging-genetic patterns (From ABCD, see Figs 1 and 2) with atrophy and disease epicentre maps from IGE and six common psychiatric disorders, specificity analyses showed that spatial correlations between PRS-HS and TLE-HS effects (see Figs 3 and 4) were statistically among the highest, even when compared against the different conditions (Table 1; IGE: Supplementary Fig. 4; psychiatric conditions: Supplementary Figs 5 and 6).

**Table 1:** Spatial correlation between effects of PRS-HS and different conditions

Analysis	Correlation (*r*)	*P*-value (*P*_spin_)	Comparison to TLE-HS (*P*_L/R_)
Idiopathic generalized epilepsy (IGE)			
Regional	0.42	0.004	0.306/0.449
Functional epicentre	0.76	<0.001	0.004/0.005
Structural epicentre	0.70	<0.001	0.39/0.002
Attention deficit/hyperactivity disorder (ADHD)			
Regional	−0.29	0.071	<0.001/<0.001
Functional epicentre	−0.80	<0.001	<0.001/<0.001
Structural epicentre	−0.67	<0.001	<0.001/<0.001
Autism spectrum disorder (ASD)			
Regional	0.14	0.322	0.0751/0.133
Functional epicentre	0.84	<0.001	0.015/0.06
Structural epicentre	0.71	<0.001	0.36/0.002
Bipolar disorder (BD)			
Regional	0.08	0.328	0.014/0.039
Functional epicentre	−0.15	0.076	<0.001/<0.001
Structural epicentre	−0.33	0.011	<0.001/<0.001
Major depressive disorder (MDD)			
Regional	−0.41	0.005	<0.001/<0.001
Functional epicentre	−0.72	<0.001	<0.001/<0.001
Structural epicentre	−0.86	<0.001	<0.001/<0.001
Obsessive compulsive disorder (OCD)			
Regional	−0.13	0.171	<0.001/<0.001
Functional epicentre	−0.38	<0.001	<0.001/<0.001
Structural epicentre	−0.69	<0.001	<0.001/<0.001
Schizophrenia (SCZ)			
Regional	0.17	0.184	0.044/0.092
Functional epicentre	0.41	0.002	0.001/<0.001
Structural epicentre	0.60	<0.001	0.218/<0.001

L = left; R = right; PRS-HS = polygenic risk score for epilepsy-related hippocampal sclerosis; spin = Spin permutation-based testing; TLE-HS = temporal lobe epilepsy with hippocampal sclerosis.

### Transcriptomic associations

Structural effects of PRS-HS shared a large number of genes with atrophy distributions in left (*n*_overlapping_ = 2274, *P*_FDR_ < 0.001) and right (*n*_overlapping_ = 2264, *P*_FDR_ < 0.001) TLE-HS. Ontological enrichment of these genes revealed biological processes involved in ion transmembrane transport, synaptic signalling, and neuronal development (all *P*_FDR_ < 0.05; Supplementary Fig. 7).

### Robustness analyses

Our findings were not affected by varying the *P*_SNP_ thresholds (*n* = 7; 0.001, 0.05, 0.1, 0.2, 0.3, 0.4, 0.5) used to construct individualized PRS-HS. Across the range of predictive thresholds, widespread decreases in thickness were related to PRS-HS, with the strongest associations again in parietal and temporal regions (Supplementary Fig. 8A). Recapitulating the reliability of threshold-specific effects, we demonstrated high similarities among different thresholds (100.0% of correlations were significant, *P*_spin_ < 0.05). Moreover, we found comparable associations between imaging-genetic and cortical atrophy maps in left (89.2% of correlations were significant, *P*_spin_ < 0.05) and right TLE-HS (67.9% of correlations were significant, *P*_spin_ < 0.05; Supplementary Fig. 8B).

Translating this approach to network models of PRS-HS, temporo-limbic and parietal epicentres identified in the main analyses were consistent across different *P*_SNP_ thresholds (Supplementary Fig. 9A). The spatial distribution of these network epicentres was highly correlated with one another (100% of correlations were significant, *P*_spin_ < 0.05; Supplementary Fig. 9B).

## Discussion

Emerging literature emphasizes the importance of genotype-phenotype associations in understanding the etiological mechanisms of epilepsy. Capitalizing on recent imaging-genetic initiatives, we combined genetic risk and whole-brain anatomy to characterize the polygenic burden of epilepsy-related HS in typical development. We found widespread decreases in cortical thickness associated with elevated PRS-HS, with the greatest effects in temporal and parietal regions. These imaging-genetic correlations were anchored to the connectivity profiles of fronto-parietal and temporo-limbic epicentres, and may play a crucial role in the network vulnerability of the brain. Structural correlates of PRS-HS further mirrored case-control atrophy and network epicentres observed in patients with TLE-HS. Findings were replicable across different *P*_SNP_ thresholds as well as different epilepsy case-control studies. Taken together, PRS-associated structural vulnerabilities may represent an early biomarker for TLE-HS pathogenesis, offering new avenues for risk stratification and pre-emptive interventions based on their genetic profiles.

Structural brain organization in typical development includes a complex and genetically determined cascade of changes from childhood to adolescence and ultimately to adulthood. Cross-sectional and longitudinal characterization of cortical grey matter tissue has demonstrated global and regional thinning during this period.^53-57^ Despite being an important aspect of normal maturation, deviations from typical development have been associated with vulnerability for various neurological and psychiatric conditions,^58-60^ including TLE-HS.^61-63^ While the exact pathogenesis of TLE-HS remains unknown, genetic studies have characterized the role of common susceptibility variants in patient cases.^13-16^ These variants account for a moderate proportion of disease phenotypic variance and may have adverse effects on structural brain development.^15^ Core to our analytical framework is the association of individualized genetic risk profiling and mapping of structural brain phenotypes, pinpointing the morphological vulnerabilities influenced by underlying predisposition to the disease. Particularly relevant for a complex disorder that is affected by many small-effect variants, PRS provides a personalized and compact measure of overall genetic liability.^21-23^ Linked imaging-derived phenotypes would help visualize the structural and biological impacts of common variant accumulation.^28^ Examining a neurotypical population, we identified widespread cortical thinning in children with elevated PRS-HS, and conversely, no relationship in the hippocampus: genetic risk may not be determinant or causative of HS, but rather serve to influence the cortical alterations. These changes may reflect a predisposition to developing a network of regions with greater propensity for epilepsy. Enrichment of risk variants related to focal epilepsy has been reported in patients with early-onset seizures.^24,25^ Childhood-onset epilepsy has also been associated with widespread structural alterations extending beyond the seizure focus.^63,64^ Given that thickness changes in development reflect pruning and neuronal maturation,^65-67^ high genetic risk to TLE-HS may accelerate and alter synaptic elimination and/or strengthening, potentially promoting an epileptogenic network.^68^ Atypical structural modelling of the developing brain related to genetic risk may therefore help predict a child's susceptibility to epilepsy.

While imaging-genetic analyses indicate significant associations between PRS-HS and structural brain changes, the observed effect sizes are relatively small, in line with those reported in previous studies across different, genetically mediated conditions.^44-46,69^ It is essential to consider the context of a typically developing cohort where the genetic burden of TLE-HS is reduced. The adverse impacts of risk variants on brain structure may be more subtle than those observed in a patient population with cumulative consequences of genetic, environmental, and disease-related factors. Moreover, it is difficult to identify the predictive value of PRS-related morphological changes in disease onset without systematic long-term clinical follow-up. Ideally, the latter would have sufficient depth to determine a potential future epilepsy conversion of individuals initially deemed as neurotypical. Longitudinal patient-level data containing both genetics and imaging, prior and subsequent to disease onset, are necessary to address the pivot from PRS-related changes to clinically relevant phenotypes, but have not been collected to date on a large scale. Despite these methodological challenges, using a population-based cohort, such as ABCD, provides a starting point for detecting these relationships and improving our understanding of how genetic predispositions associated with certain clinical phenotypes correlate with brain structural vulnerabilities at the population level.

Alterations in TLE-HS commonly implicate many brain regions organized within interconnected systems.^7,9,70-75^ Understanding these interactions and their contributions to epileptogenesis requires the integration of connectome architecture. Epicentre mapping emerges as a valuable data-driven method to pinpoint critical regions—termed epicentres—that may serve as critical anchors in the manifestation of common genetic variants.^9,76-78^ Analysing how localized genetic vulnerabilities propagate through distributed brain regions can identify potential network pathways that link genetic risk to pathological mechanisms. In particular, marked PRS-related thinning occurs in regions strongly connected to temporo-limbic and parietal territories. Diffusion MRI is highly effective at detecting long-range fibre bundles and direct monosynaptic structural connections, but it does not fully capture short-range intracortical and spatially distributed polysynaptic cortical systems.^79^ By contrast, resting-state functional MRI can detect functional connectivity in the absence of direct structural connections, and thus is more informative about polysynaptic configurations.^80,81^ These temporo-limbic and parietal epicentres are characterized by a disproportionately high number of mono- and polysynaptic connections and serve as crucial areas for the integration and signal broadcasting across different structural and functional networks. Consequently, such regions are inherently vulnerable to TLE-HS pathology.^9,74,82^ Given the convergence between functional and structural genetic epicentres, these regions also show susceptibility to the effects of accumulated genetic risk factors. Local changes related to PRS-HS may therefore disrupt global network organization, such that it increases vulnerability to targeted hub attacks, and potentially to seizure activity. The spatial and system-level context provided by these imaging-genetic associations—beyond PRS alone—may help identify vulnerable circuits for enhanced monitoring and neuromodulatory therapeutics.^83^

To bridge the transition from genetic vulnerability to clinical phenotype, we contextualized regional and network correlates of PRS with case-control atrophy and epicentres, revealing strong spatial resemblance: thinner areas in children with elevated genetic risk tend to be thinner in patients and be highly connected to disease-related networks. Structural alterations have been consistently identified in TLE-HS, and are most marked in mesiotemporal, limbic, and sensorimotor areas.^3-7,83^ These alterations are also anchored to the connectivity profiles of distinct temporo-limbic and parietal epicentres.^9^ While family-based studies have shown low heritability for these atrophy patterns in healthy relatives,^84-86^ these predisposed regions may be too subtle and difficult to capture in endophenotype paradigms due to the complexity of epilepsy. Large sample sizes with varying genetic risk, as utilized herein, are required to characterize these imaging-genetic associations.^87^ In combination with disease contextualization, we found a common driving process between genetic risk manifestations and disease effects. The polygenic burden of TLE-HS may therefore impact biological mechanisms—neuronal signalling, ion transport and neurodevelopmental pathways, as identified in transcriptomic associations—underlying brain structure and network architecture, and potentially influence disease vulnerability and pathogenesis. Although insufficient to cause TLE-HS alone due to its multifaceted components, genetics may increase susceptibility to the consequences of external factors^20,88^ in vulnerable regions and their networks through specific biological pathways.

Imaging-genetic associations also mirrored IGE-related atrophy and epicentres, to a lesser extent than TLE-HS. Pleiotropy—whereby a genetic variant influences multiple traits–occurs in the genetics of complex traits and disorders.^89,90^ Relevant to epilepsy, certain genetic variants may contribute to the vulnerability to both generalized and focal syndromes.^15^ Despite the wide clinical spectrum of epilepsy, the shared genetic architecture may play a role in some common pathological features.^91^ Supported by literature demonstrating similar patterns of cortical thinning across different subtypes,^7^ our imaging-genetic model further adds to a common structural signature, such that widespread atrophy may originate from shared genetic pathways and reflect a more general epilepsy-related phenomenon. Similarly shown with disease epicentres herein, such a concept may also translate to network-level alterations. These associations may be potential biomarkers and encourage further exploration of the shared and trait-specific effects of common genetic factors in TLE-HS and the broader spectrum of epilepsy.

Specificity of these associations was supported by the fact that spatial correlations between imaging-genetic effects and disease effects in TLE-HS ranked the highest compared to several common psychiatric disorders. Many neurological and psychiatric conditions exhibit converging spatial patterns of cortical changes and network profiles commonly colocalizing to higher-order transmodal regions, which are known to serve as epicentres of network organization and vulnerability.^92,93^ Regional pathological processes might propagate from common disease epicenters to connected brain regions, leading to network-spreading patterns of cortical alterations.^9,76-78,94-96^ These centrally located areas in the network are therefore particularly vulnerable to pathophysiological perturbations and may explain the statistical significance of multiple correlations in our analyses. However, the consistent and greater associations of PRS-HS with TLE-HS suggest that—despite the broad involvement of distributed brain networks—there may be a disease-specific signature in the imaging-genetic associations that reflects meaningful biological specificity.

Limitations of imaging-genetic associations with respect to the GWAS-identified SNPs need to be highlighted. Firstly, summary statistics used for PRS calculation were based on GWAS of ‘focal epilepsy with documented HS’.^15^ Although it represents the most common pathological substrate for TLE-HS, hippocampal alterations occur in other epilepsy syndromes, and may be a cause, or consequence of epilepsy, or both.^10,97,98^ This phenotypic heterogeneity may impact the genetic associations identified. A more accurate delineation is crucial for detecting variants related to TLE-HS and its downstream effects, which may not be fully captured in our PRS correlations. Secondly, the same GWAS was mainly conducted in individuals of European ancestry.^15^ While our findings may be specific to European populations, they may not generalize to other under-represented groups.^99^ Replication of imaging-genetic effects, particularly using a GWAS that includes larger and more diverse cohorts—ideally with inclusion criteria that specifically define TLE-HS—could enhance the reliability and generalizability of imaging-genetic effects. This would improve the power to detect smaller effect sizes and refine the understanding of how specific genetic variants influence brain structure.

In summary, the present work highlights the potential for applying imaging-genetic frameworks to uncover the interplay between genetic predisposition, neuroanatomical changes, and epilepsy pathogenesis. Structural vulnerabilities linked to high PRS-HS in childhood resembled atrophy and epicentres commonly observed in patients. Collectively, these results highlight important candidates for stratification efforts that can unravel the complex aetiology of epilepsy, advancing the use of PRS as a potential biomarker for disease risk and for developing targeted interventions that prevent or limit progression of epilepsy.

## Supplementary Material

awaf259_Supplementary_Data

## Data Availability

Genotyping and imaging data is available from the ABCD study upon application through NIMH Data Archive (https://nda.nih.gov/). GWAS summary statistics are available at http://www.epigad.org/gwas_ilae2018_16loci.html. The HCP dataset is available at https://db.humanconnectome.org/. Neuroimaging data from the ENIGMA (meta-analysis of summary statistics) are available for download (https://github.com/MICA-MNI/ENIGMA).

## Appendix 1

Additional members of ENIGMA Consortium Epilepsy Working Group: Eugenio Abela, Julie Absil, Saud Alhusaini, Sarah J. A. Carr, Gianpiero L. Cavalleri, Esmaeil Davoodi-Bojd, Norman Delanty, Chantal Depondt, Colin P. Doherty, Martin Domin, Sonya Foley, Aoife Griffin, Graeme D. Jackson, Magdalena Kowalczyk, Angelo Labate, Soenke Langner, Mario Mascalchi, Pascal Martin, Mark P. Richardson, Christian Rummel, Mira Semmelroch, Mariasavina Severino, Aditi Singh, Rhys H. Thomas, Manuela Tondelli, Domenico Tortora, Felix von Podewills, Sjoerd B. Vos, Christopher D. Whelan, Roland Wiest, Junsong Zhang.
